# How Degeneration of Cells Surrounding Motoneurons Contributes to Amyotrophic Lateral Sclerosis

**DOI:** 10.3390/cells9122550

**Published:** 2020-11-27

**Authors:** Roxane Crabé, Franck Aimond, Philippe Gosset, Frédérique Scamps, Cédric Raoul

**Affiliations:** 1 The Neuroscience Institute of Montpellier, INSERM, UMR1051, University of Montpellier, 34091 Montpellier, France; roxane.crabe@inserm.fr (R.C.); franck.aimond@inserm.fr (F.A.); philippej.gosset@inserm.fr (P.G.); frederique.scamps@inserm.fr (F.S.); 2 Laboratory of Neurobiology, Kazan Federal University, 420008 Kazan, Russia

**Keywords:** amyotrophic lateral sclerosis, spinal cord, cortex, motoneuron, astrocytes, interneuron, oligodendrocytes, degeneration

## Abstract

Amyotrophic lateral sclerosis (ALS) is a fatal neurological disorder characterized by the progressive degeneration of upper and lower motoneurons. Despite motoneuron death being recognized as the cardinal event of the disease, the loss of glial cells and interneurons in the brain and spinal cord accompanies and even precedes motoneuron elimination. In this review, we provide striking evidence that the degeneration of astrocytes and oligodendrocytes, in addition to inhibitory and modulatory interneurons, disrupt the functionally coherent environment of motoneurons. We discuss the extent to which the degeneration of glial cells and interneurons also contributes to the decline of the motor system. This pathogenic cellular network therefore represents a novel strategic field of therapeutic investigation.

## 1. Introduction

Amyotrophic lateral sclerosis (ALS) is an adult-onset neurodegenerative disease characterized by the selective and progressive loss of upper and lower motoneurons. ALS begins with focal muscle weakness and wasting that relentlessly spreads to other body parts, leading to death mostly from respiratory failure within three years of onset. ALS is predominantly a sporadic disease, although at least 10% of cases are due to inherited mutations. Among the 50 genes that have been linked to ALS, pathogenic mutations in *chromosome 9 open reading frame 72*, *superoxide dismutase-1* (*SOD1*), *fused in sarcoma* (*FUS*), and *TAR DNA binding protein* (encoding TDP-43) genes are most frequently found [1]. Numerous studies using a wide spectrum of genetic ALS models have shed light on potential intrinsic and extrinsic factors responsible for the susceptibility of motoneurons to the disease. The cell-autonomous mechanisms that ground motoneuron vulnerability are associated with calcium (Ca^2+^) mishandling, susceptibility to endoplasmic reticulum (ER) stress and death receptor signaling, modified electrophysiological properties, and/or altered RNA homeostasis [2,3,4,5]. Non-neuronal cells, including astrocytes, oligodendrocytes, microglial cells, and blood-derived immune cells, can contribute to the selective degeneration of motoneurons through the release of inflammatory mediators and cytokines, production of reactive oxygen species, loss of homeostatic functions associated with metabolic supply, anti-inflammatory factors, glutamate clearance and neurotrophic support, in addition to direct killing [6,7,8,9]. The status and inflammatory response of cells constituting the local environment of motoneurons have received the most attention. However, the degeneration of astrocytes, oligodendrocytes, and neurons with local (and long-range) connectivity has been documented as events that precede or are concomitant with motoneuron death. Loss of motoneuron neighboring cells might therefore be regarded not only as a consecutive dismantling of cells partaking in the network ensuring motoneuron function but also as an active component of ALS pathogenesis. Our goal is to review the current understanding of neurodegenerative processes that occur in the vicinity of motoneurons and take part in the deterioration of motor functions. We suggest not characterizing ALS as an exclusively motoneuron disorder, and consider these grey areas of degenerative events to stimulate further investigation and develop new efficient approaches to treat the disease.

## 2. The Degenerative Astrocytes

### 2.1. Characterisation of AbGC in ALS Pathogenesis

**Figure 1:**
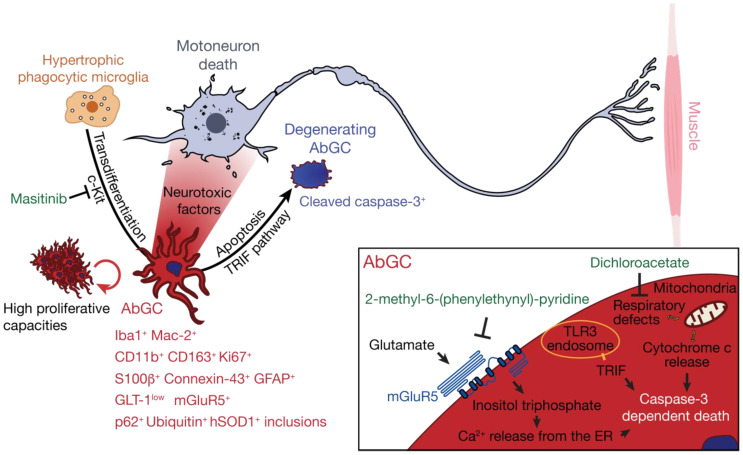
AbGC are highly toxic toward motoneurons, and present cellular dysfunctions ultimately leading to their degeneration. Hypertrophic phagocytic microglia in the motoneuron microenvironment can transdifferentiate, possibly in a c-kit-depend manner, towards an aberrant astroglial phenotype (in red) that can be identified by a combination of markers in amyotrophic lateral sclerosis (ALS). AbGC show defects in mitochondrial functions, glutamate handling, and high proliferative capacities and produce soluble factors that are particularly toxic for motoneurons. Several intracellular events lead to AbGC death, including activation of the TLR3-TRIF pathway, inositol triphosphate formation and Ca^2+^ release from internal store, cytochrome c release from mitochondria, and caspase-3 activation. In green are indicated small molecules that, by targeting AbGC-associated pathogenic mechanisms, confer therapeutic benefits in ALS mice.

Astrocytes represent one of the most abundant cell types in the central nervous system (CNS) and support numerous physiological functions that include trophic and metabolic support of neurons, regulation of synapse formation and activity, ion homeostasis, and induction and maintenance of the blood–brain barrier. A key event in the physiopathology of neurological disorders is the neuroinflammatory response mediated by astrocytes, whose functions can drastically change. Reactive astrogliosis is a salient feature that was initially documented in histopathological examinations of the spinal cord and motor cortex of patients with ALS [10,11]. Subsequently, a compelling body of evidence has been established that suggests astrocytes contribute through different mechanisms to the non-cell-autonomous degeneration of motoneurons [7,12]. The toxicity of ALS astrocytes towards motoneurons was shown to be mediated by soluble factors that include extracellular vesicles, tumor necrosis factor alpha, interferon gamma (IFNγ), nerve growth factor, and reactive oxygen/nitrogen species [13,14,15,16,17,18]. Degeneration of astrocytes, which is another manifestation of astrocytic pathology, was first evidenced in the spinal cord of mutant SOD1 mouse as ubiquitin-, activated caspase-3-, and SOD1-positive round inclusions delineated by glial fibrillary acidic protein (GFAP) [19]. This population of degenerating astrocytes was also referred to as spheroid GFAP-positive cells (SGPCs) due to the unusual morphology of the cells, which are composed of a round hypertrophic cell body and thick short processes [20]. A few years later, Luis Barbeito and colleagues shared their observations on phenotypically aberrant astrocytes, referred to as aberrant astrocytes (Aba), which, once isolated from the spinal cord of ALS symptomatic rats, were able to proliferate abundantly in culture [21]. Aba were also demonstrated in the tissue where they take the form of hypertrophic reactive astrocytes. Although the parallel between both groups’ studies has never been clearly established, the description of these specific astrocyte populations shows great similarity in localization, distinctive histological features, fate, and function. In accordance with the latest findings and terminology [22], we decided to use the term aberrant glial cells (AbGC) when referring to the generic concept of aberrant degenerating astrocytes in ALS. AbGC are found in the close vicinity of motoneurons; 93% of are located less than 130 µm from a motoneuron. When considering molecular markers, Aba cells strongly express the astrocytic proteins S100β and connexin-43 [21], and harbor, like SGPCs, an unusual blurred and annular-shaped GFAP staining, restricted to the cell body periphery and proximal processes. Consistent with their active proliferation and degenerating state, AbGC are Ki67^+^ and cleaved-caspase-3^+^. In addition, the cells present inclusions of human SOD1, p62, and ubiquitin [19,23], again demonstrating their abnormal state in vivo. Unexpectedly, AbGC also express several markers of the macrophage/microglial lineage. We can, however, note a difference in the microglial markers expressed by AbGC in the symptomatic SOD1^G93A^ rats compared to their mouse equivalent. Whereas rat AbGC express Iba1, CD163, Cd11b, and CD68 [24], mouse AbGC are CD68 and Iba1 negative but express Mac-2, a marker of phagocytic microglia [23,25,26] (Figure 1). Even if surprising, the chimeric phenotype of AbGC is not the first case of cells sharing microglial and astrocytic markers, as seen in patients with glioblastoma multiforme and cerebral ischemia models [25,27,28]. In vitro studies have shown that AbGC actually originate from activated microglial cells [21,24]. Although anecdotal in vivo at the asymptomatic stage, this astrocyte/microglial phenotype is predominant in the motoneuron microenvironment at the symptomatic stage, with Iba1-expressing cells representing up to 70% of AbGC in ALS rats [21,24]. In mice, the surge of these AbGC was evaluated to be 21-fold at onset and 57-fold at end stage [20]. Given the great similarities between the above-mentioned research, it is likely that the observations of both teams come from the study of the same subpopulation of astrocytic cell, possibly originating from aberrant microglia in the ALS rat and mouse.

### 2.2. AbGC are Dysfunctional Degenerating Cells 

Extensive proliferation is one of the key characteristics of AbGC. In addition, cultured AbGC are not subjected to replicative senescence, because they survived to a year of successive passage, nor to contact inhibition [21,29]. Interesting results were obtained with masitinib, an inhibitor of c-Kit, and a member of the type III receptor tyrosine kinase family located upstream of pathways controlling proliferation, differentiation, and migration of hematopoietic cells [30]. Chronic masitinib administration more than halved the number of hypertrophic phagocytic microglia transdifferentiating into AbGC, in addition to their pro-inflammatory profile and their ability to migrate [31]. Remarkably, the treatment reduced motoneuron loss and extended life expectancy of ALS mice.

Mitochondrial damage is one of the many subcellular dysfunctions affecting AbGC. Mitochondria of AbGC display reduced length, altered morphology, and major impairments of the respiratory functions compared to wildtype and *SOD1^G93A^* astrocytes [22,29]. Dichloroacetate (DCA) is an inhibitor of the pyruvate dehydrogenase kinase that reorients pyruvate consumption towards mitochondrial oxidative phosphorylation. Treatment with DCA is able to restore mitochondrial functions in AbGC cultures, in addition to slowing their proliferation and reducing their neurotoxicity towards motoneurons [22]. This drug, which has been on the market for more than 40 years, can cross the blood–brain barrier and its chronic administration has been shown to be effective on the disease course, to increase motoneuron survival, and reduce glial reactivity and the number of AbGC in the ventral horn of the spinal cord [22,32].

Glutamate induced-excitotoxicity is one of the putative causes of motoneuron loss in ALS. This is mainly supported by the observation of a loss of the major glutamate transporter GLT-1 expression by astrocytes, making them unable to properly buffer the glutamate surplus from motoneuron excitatory inputs. Early studies in the asymptomatic *SOD1^G93A^* mouse revealed that AbGC express low levels of GLT-1 [20,21,33]. Two studies also suggested that glutamate is closely linked to the degenerative state of AbGC, which express the metabotropic glutamate receptor 5 (mGluR5) as shown in the spinal cord of sporadic ALS patients and *SOD1^G93A^* mice [20,33]. In the spinal cord of *SOD1^G93A^* mice, levels of mGluR5 increase before disease onset and then decrease to reach levels comparable to control mice [33]. The exposure of SOD1 mutant astrocytes to glutamate selectively triggers their death through mGluR5 signaling, which implicates the production of inositol trisphosphate and release of Ca^2+^ from the ER store [20,33]. Administration of 2-methyl-6-(phenylethynyl)-pyridine, a selective mGlurR5 antagonist, in *SOD1^G93A^* mice significantly reduced the number of cleaved-caspase-3^+^ astrocytes, although there was no difference in the total number of AbGC, delayed onset, and prolonged survival [20] (Figure 1).

One consensus concerning AbGC, evidently linked to the many deficiencies above-mentioned, is their degenerative state and consequent death through apoptosis [19,20,23]. The study in mice revealed that the first signs of astrocyte degeneration occurred around disease onset with 18% of cleaved-caspase-3^+^ AbGC and reached 33% by end stage [20]. Activated caspase-3 is able to digest the GFAP cytoskeleton into a 20 kDa fragment found in the homogenate of spinal cord from ALS mice [20], which could explain the characteristic blurred GFAP immunoreactivity of AbGC in vivo and their peculiar spheroid morphology. Absence of intermediate filament structures in AbGC in vitro could suggest a near total digestion of GFAP following caspase activation [29]. Interestingly, the apoptosis rate of AbGC can be reduced by the genetic ablation of the TIR domain-containing adaptor inducing interferon-β (TRIF) [23]. TRIF is an adaptor molecule in the Toll-like receptors (TLR) 3 and 4 signaling cascades. *SOD1^G93A^* mice with a targeted deletion of the *Trif* gene present an increased number of Mac-2^+^ GFAP^+^ AbGC in the ventral horn of the spinal cord with a reduced proportion of cleaved-caspase-3^+^ AbGC compared to *SOD1^G93A^* controls. Apoptosis induced in a TRIF-dependent pathway was therefore proposed as a means to reduce deleterious effects of AbGC on motoneurons [23].

### 2.3. AbGC are Highly Toxic Towards Motoneurons and Aggravate Disease Progression in ALS Rodent Models

A wealth of studies have demonstrated that ALS mutant astrocytes are selectively toxic to motoneurons through the production of soluble factors [7]. In the first study on AbGC, co-culture experiments demonstrated that these cells are far more toxic towards motoneurons than *SOD1^G93A^* neonate-derived astrocytes, allowing less than 10% survival (compared to 60% with regular mutant astrocytes). This toxicity implicates the secretion of toxic factors, as evidenced by the use of condition media of AbGC that display a motoneuron-selective toxicity [21]. To further explore the extent of the pathogenic potential of AbGC, transplantation experiments have also been performed in the lumbar spinal cord of wildtype rats [34]. Transplanted AbGC are able to proliferate and potently provoke bilateral microglial and astroglial activation compared to rats injected with vehicle or *SOD1^G93A^* neonatal microglia. Transplanted AbGC did not migrate along the rostrocaudal axis but elicited a strong gliosis up to the cervical segments of the spinal cord. Such a phenotype is coherent with the release of toxic/pro-inflammatory soluble factors by AbGC. Notably, the authors indicate that even though motoneuron survival is comparable to controls seven days after transplant, the presence of cytoplasmic ubiquitin aggregates could be an early sign of motoneuron degeneration. The experiments never exceeded seven days, which could have been insufficient to elicit noticeable motoneuron death. Overall, data collected from experimental models during the past two decades strongly suggest that AbGC play an important role in non-cell-autonomous mechanisms leading to motoneuron degeneration in ALS.

Additional evidence of astrocyte death is provided by the study of human astrocytes derived from induced pluripotent stem cells (iPSCs) that were established from an ALS patient harboring a mutation in TDP-43 [35]. A cell-autonomous mechanism of astrocyte degeneration associated with TDP-43 mislocalization was here described as likely occurring in a caspase-independent manner. A similar cell-autonomous mechanism affecting astrocyte survival was observed in iPSC-derived valosin-containing protein-mutant astrocytes [36]. Very recently, it was shown that the release of fragmented mitochondria from SOD1^G93A^-expressing microglial cells induces the inflammatory A1 type of astrocyte [37]. A1 is a particularly neurotoxic population of reactive astrocytes [38], as are AbGC. The activated microglia lead to mitochondria dysfunction and fragmentation, reactive oxygen species and proinflammatory cytokine production, and death of naive primary astrocytes [37]. Beyond demonstrating that astrocyte degeneration is not a consequence of an idiosyncratic effect of SOD1, it also raises additional questions about the link between A1 type and AbGC, in addition to cell- and non-cell autonomous mechanisms that result in this astrocytic pathology.

## 3. Oligodendrocyte Degeneration

**Figure 2:**
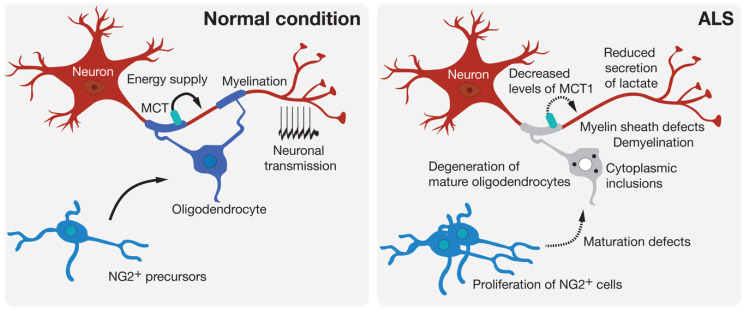
Oligodendroglial pathology in ALS. Oligodendrocytes can contribute to neuronal function by providing metabolic support and potentiating signal conduction by myelination of the axon. Many signs of oligodendrocyte dysfunction and loss can be observed prior to motoneuron degeneration in experimental models and in patients with ALS.

Oligodendrocytes are found throughout the entire CNS where their most known role is the myelination of axons. Oligodendrocytes produce the myelin sheath that acts as an electrical insulator to facilitate conduction in axons. Oligodendrocytes also ensure basic functions in providing trophic and metabolic support to neurons [39]. Thus, oligodendrocytes are able to transform glucose into lactate or pyruvate and supply these products to neurons via monocarboxylic acid transporters (MCT) [40] (Figure 2). Increasing evidence shows that oligodendrocytes contribute to many neurodegenerative diseases through mechanisms related to demyelination or the metabolic support provided to neurons [41]. Loss of myelin is observed in the gray matter of the cortex and the ventral part of the spinal cord of patients with ALS [42]. Mature oligodendrocytes that are found in the gray matter of the spinal cord of SOD1^G93A^-expressing mice degenerate before the loss of motoneurons and the first symptoms of the disease [8,42]. This degeneration is followed by the proliferation of NG2^+^ progenitor cells of oligodendrocytes in the spinal cord. However, these NG2^+^ cells do not mature and are therefore unable to replace the pool of already degenerated oligodendrocytes, thus leaving axons of motoneurons demyelinated. In mice, the genetic deletion of mutated SOD1 in oligodendrocyte precursors markedly delayed disease onset and prolonged the survival of the mice [42]. More recently, a study performed on zebrafish, in which only mature oligodendrocytes expressed an ALS-linked mutated SOD1, showed an increased proliferation of oligodendrocyte precursors and an increased degeneration of mature oligodendrocytes. These events were followed by neuromuscular junction defects and the degeneration of motoneurons [43], and were accompanied by behavioral abnormalities, including anxiety-like behavior, learning impairment, and motor defects. The expression of SOD1^G93A^ or TDP-43^Q331K^ in mature oligodendrocytes leads to disturbances in myelin organization that might affect axonal conductance [43]. Another noxious effect of oligodendrocyte pathology on motoneuron function is linked to the delivery of lactate, the major energy source, through MCT-1. Indeed, an important reduction in MCT-1 levels can be observed in sporadic ALS patients and *SOD1^G93A^* mice [44]. The susceptibility of motoneurons to human oligodendrocytes differentiated from iPSCs of ALS patients was associated with reduced levels of MCT-1 and lower secretion of lactate [45] (Figure 2).

Post mortem tissue analyses of ALS patients have shown that TDP-43 and FUS form cytoplasmic inclusions in oligodendrocytes [46,47,48]. The phosphorylated form of TDP-43, which is prone to form ubiquitin-positive inclusions [49], is present in the primary cortex and spinal cord of ALS patients [47,48]. The cytoplasmic accumulation of TDP-43 aggregates can result in a loss-of-function via the progressive depletion of TDP-43 from the nucleus [50]. With regards to this mechanism, the conditional deletion of TDP-43 transgene in myelinating oligodendrocytes leads to the deterioration of motor scores and reduced lifespan [51]. The depletion of TDP-43 in mature oligodendrocytes is accompanied by a reduction in myelin, myelin sheath defects, and myelination capacities in the CNS. TDP-43 depletion causes a marked gray matter oligodendrocyte death by necroptosis, which can be compensated by an enhanced proliferation of NG2^+^ oligodendrocyte precursor cells. However, there appears to be no effect on the number and size of motoneurons [51]. The pathogenic mislocalization of FUS in the cytoplasm leads to an increased number of white matter oligodendrocytes in the spinal cord, which occurs independently of motoneuron alteration. These oligodendroglial defects in the spinal cord are corroborated by the downregulation of genes implicated in myelination [52]. Oligodendrocytes therefore represent a predominant site of cytoplasmic inclusions, as also evidenced in the CNS of ALS patients by the accumulation of p62, a cargo receptor for ubiquitinated proteins [53], or the Von Hippel Lindau protein, involved in the degradation of fragmented TDP-43 [54].

The dysfunction and degeneration of oligodendrocytes is, in conclusion, an important factor damaging the functional unity of the motor system in the disease (Figure 2). However, mechanisms underlying their degeneration remain elusive, but could involve the excitotoxicity mechanism induced by α-amino-3-hydroxy-5-methyl-4-isoxazolepropionic acid/kainate glutamate receptors [55], the pro-inflammatory cytokine IFNγ [56], which was previously described as an astrocyte-derived motoneuron toxic factor and whose levels are increased in the spinal cord of ALS patients and mice [15,57], or the disruption of protein homeostasis in respect of cytoplasmic inclusions observed in the disease, because impairment of the unfolded protein response can lead to death of mature oligodendrocytes in mice [58].

## 4. Interneuronopathy in ALS

### 4.1. Inhibitory Interneuron Degeneration

Central pattern generators (CPGs) are the basic neuronal circuits responsible for the fine control of skilled movements and the general coordination of locomotion. Interneurons are one of the main regulators of neuronal signaling and play an important function in the spinal CPG network. The coordinated activity of several interneuron types, such as inhibitory neurons, plays a crucial role in the modulation of motoneurons output to the appropriate muscles. At least 11 neuronal types of interneurons have been identified and inhibitory interneurons mainly use γ-aminobutyric acid (GABA) or glycine as neurotransmitters. These neurotransmitters are released from V1, V0_D_, and V2b interneurons, classified according to their respective combinatorial transcription factor code. The V1 class includes Ia inhibitory interneurons and Renshaw cells, V0_D_ are commissural interneurons, and V2b are primarily ipsilateral with a subset with commissural projections [59,60,61]. The circuitry complexity of the pyramidal upper motoneurons that is located in layer 5 of the motor cortex is illustrated by the diversity of projection targets; long-distance apical dendrites; the diversity of inputs including those coming from local circuitry, cortical layer 2/3, thalamocortical neurons, or callosal projection neurons; and the large heterogeneity of GABAergic inhibitory neurons classified in 3 major groups according to expression of parvalbumin, somatostatin, and serotonin receptor 5HT3aR [62,63,64,65].

**Figure 3:**
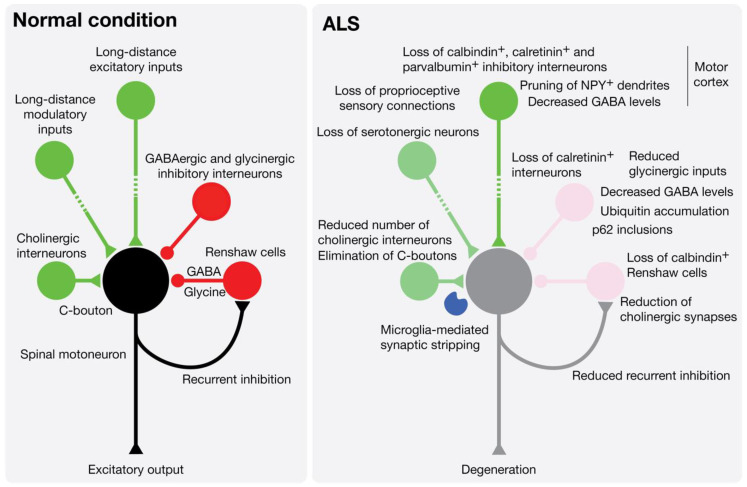
Interneuron pathology leads to an impaired excitation/inhibition balance in ALS. Lower motoneurons execute voluntary movements by integrating central descending and peripheral commands, and local inhibitory (GABAergic or glycinergic interneurons and Renshaw cells) and modulatory (cholinergic and serotonergic neurons) inputs. During the natural course of the disease, including prior to the first signs of clinical symptoms, dysfunctions and loss of the different classes of interneurons have been documented in the motor cortex and spinal cord of ALS patients and mouse models of the disease.

Transcranial magnetic stimulation in ALS patients showed a reduced intracortical inhibition that suggests an impairment or loss of inhibitory interneurons [66,67,68,69,70]. A decreased density of neurites positive for neuropeptide Y (NPY), a neurochemical marker of inhibitory interneurons, was found in the motor cortex of ALS patients. Although the total number of NPY^+^ interneurons was not affected, they were found to be atrophic with signs of dendritic pruning [71]. Post mortem analysis of ALS patient spinal cord indicated a loss of dorsomedial interneurons [72], and a loss of calbindin^+^ interneurons and their processes in the ventral horn [73]. In more specific terms of the interneuronal identity, the density of GABAergic interneurons was found to be reduced in the cortical layer 5 of the primary motor cortex of ALS patients [74]. Another study showed a decrease in parvalbumin^+^ GABAergic interneurons in the motor cortex of ALS patients [75]. These results can be correlated with the decreased levels of GABA that were observed in the motor cortex of ALS patients using proton magnetic resonance spectroscopy [76]. Correspondingly, reduced volumes of distribution of [^11^C] flumazenil, a specific type of A GABA (GABA_A_) benzodiazepine receptor antagonist, were observed in the motor and premotor cortex (and in extramotor areas) of ALS patients by positron emission tomography [77] (Figure 3).

Similar results were obtained in mouse models of ALS. A decrease in calretinin^+^ interneurons was observed in the cerebral cortex and hippocampus of *SOD1^G93A^* mice [78,79]. In the spinal cord of G86R mutant SOD1 mice, a 40% loss of calretinin interneurons was observed at the symptomatic stage [80]. High-resolution magnetic resonance spectroscopy also showed a steady decrease in GABA in the spinal cord of *SOD1^G93A^* mice from pre-symptomatic to late disease stage [81]. In mice expressing TDP-43^A315T^, an ubiquitin accumulation has been observed in spinal interneurons [82], and impairments in GABAergic transmission, implicating somatostatin and parvalbumin interneurons, have been demonstrated to contribute to cortical hyperexcitability, excitotoxicity, and in fine degeneration of pyramidal neurons [83]. A study of a mutant SOD1 zebrafish model revealed that the earliest affected neurons were inhibitory interneurons, leading to a reduction in glycinergic inputs and then defects in motoneurons [84]. Electrophysiological recordings performed in spinal cord organotypic cultures, and cortical and brainstem slices of pre-symptomatic ALS mice, demonstrated an hyperexcitability, possibly due to a decrease in inhibitory neurotransmission, that could in fine lead to degeneration [85,86]. Similarly, GABA-induced release of glutamate from gliosomes isolated from the spinal cord of *SOD1^G93A^* mice was found to be enhanced [87]. In cultured motoneuron from ALS mice, a specific desensitization of GABA_A_R with α1 subunit was described [88]. In addition, a decrease in tonic GABAergic inhibition, related to a reduction in the vesicular GABA transporter [89], and a 72% reduction in GABA receptor-mediated inhibitory currents, were also described in motor cortex slices of the wobbler mouse model [90].

Renshaw cells, which are GABAergic and glycinergic inhibitory cells, receive input from motor axon collaterals and synapses, in turn, on the soma of motoneurons for negative-feedback. A loss of calbindin^+^ Renshaw cells and glycinergic synapses on motoneurons has been observed since pre-symptomatic age, prior to motoneuron loss, in spinal cord of SOD1 mutant mice [91]. Most recent evidence also favors an early disconnection of Renshaw cell feed-forward inputs from motoneurons [92]. The early loss of glycinergic interneurons or the Renshaw cell-mediated recurrent inhibitory pathway can lead to motoneuron hyperexcitability. Interestingly, another study that also reported a reduced number of Renshaw cells in *SOD1^G93A^* spinal cord showed a marked reduction in cholinergic synapses on Renshaw cells, suggesting a decrease in recurrent inhibition [92,93]. This reduced recurrent inhibition was also observed in patients with ALS [94]. These results may explain how Renshaw cell alterations contribute to impairment of the excitatory/inhibitory balance and motor system coherence.

The molecular mechanisms leading to the dysfunction of interneuron inhibition and the death of interneurons remain enigmatic. It is noteworthy that transplantation of spinal progenitors, derived from iPSCs obtained from sporadic ALS patients, into the spinal cord of immunodeficient mice, leads to the predominant production of astrocytes with reactive properties and death of neurons before motoneuron degeneration. This was accompanied by a significant loss of GABAergic and glycinergic inhibitory inputs on motoneuron soma [95]. The interneuron pathology, revealed by the formation of p62^+^ skein-like inclusions in parvalbumin^+^ neurons in late stages of the disease, and which is delayed by the selective inhibition of autophagy in motoneurons, opens new fields of investigation [96].

### 4.2. Cholinergic Synapse Defects

The spinal cholinergic synapse, also called C-bouton, originates from V0c neurons, a small population of Pitx2^+^ interneurons, which is located in the lamina X that surrounds the central canal [97,98]. C-boutons form large clusters at the soma and proximal dendrites of alpha-motoneurons in the spinal cord [99]. It is now well established that C-boutons increase the firing rate of motoneurons and are involved in high task demands that recruit fast-fatigable and fatigue-resistant motor units [98,99,100]. V0c interneurons receive synaptic inputs from descending serotonergic pathways, local or long-distance vesicular glutamate transporter 2 projections, V2b inhibitory interneurons, interneurons of the dorsal horn involved in nociception, and direct input from parvalbumin^+^ non-proprioceptive sensory neurons [98,101,102]. Of note, motoneurons that innervate fast-twitch muscles and that are the most vulnerable to ALS have a greater number of C-boutons than those innervating slow-twitch motoneurons and that are largely resistant to neurodegeneration [103]. In addition, the muscarinic stimulation is motoneuron-type dependent with a higher efficacy in motoneurons vulnerable to the disease, further supporting some specific role of tasks related to C-boutons in ALS [104].

A pioneer study documented a severe loss of cholinergic synapses on motoneurons of sporadic ALS patients [105]. In two different SOD1 mutant mice, it was shown that although the number of C-boutons does not change until the symptomatic stage, thereafter a significant decrease in C-boutons per motoneuron can be observed [106]. However, a marked decrease in cholinergic synapse was observed in asymptomatic SOD1 mutant mice [93]. A detailed longitudinal analysis in *SOD1^G93A^* mice described several changes in the morphology, number of C-boutons, or M2 muscarinic receptor levels that start at pre-symptomatic stages [107]. Importantly, a loss of Lamina X cholinergic interneurons has been observed in the symptomatic ALS mouse [107], while a decrease in their choline acetyl transferase (ChAT) content was noticeable at the asymptomatic stage [93]. Additional studies show an enlargement of C-boutons in ALS mice [108,109], although the increase in the size, but not the number, was observed only in males in another report [110]. Two other studies reported a reduction in C-bouton number and density only at the end-stage of the disease [111,112] (Figure 3).

The contribution of dysfunction and loss of interneuronal cholinergic transmission in ALS remains still largely unknown. The silencing of C-boutons in *SOD1^G93A^* mice with the conditional deletion of *Chat* in Dbx1^+^ V0 interneurons accelerates the locomotor defects [113]. Moreover, decreasing the excitability of fast-twitch motoneurons through the Ca^2+^-activated chloride channel TMEM16F at C-boutons reduced stress of motoneuron and denervation at the neuromuscular junction, and maintained muscle strength [104]. To date, it remains to be demonstrated whether the loss of C-boutons is a compensatory or an aggravating mechanism of ALS, and whether the circuitry controlling V0_C_ interneurons activity is affected in ALS. An appealing pathogenic mechanism is related to the potential role of the neuroinflammatory response that accompanies motoneuron degeneration, where the stripping of cholinergic synapses in the spinal cord was proposed to be mediated by microglial cells [93,112,114]. Indeed, expression of neuregulin-1 at the post-synaptic site of C-bouton might promote the recruitment and activation of microglia [114].

## 5. Conclusions

As shown in this review, prior to or concomitant with motoneuron degeneration, deficits in glial and neuronal functions take part in the pathogenic process leading to ALS. Here, we focused on the populations of cells that participate in the local network of motoneurons and whose impairment and loss will lead to the demise of the motor system. However, a significant number of studies report that other peripheral and central neuronal types are also affected in ALS. The degeneration of serotonergic neurons in the brainstem has been described in ALS patients [115]. In SOD1 mutant mice, the loss of serotonergic neurons was evidenced at disease onset, and was associated with reduced serotonergic innervation on spinal motoneurons and the development of spasticity [116]. Additionally, in the neuromodulatory system, the loss of tyrosine hydroxylase dopaminergic neurons was reported in the substantia nigra of ALS patients [117], and was corroborated by nigrostriatal dopamine deficits [118,119]. Reduced levels of dopamine in the midbrain were also reported in *SOD1^G93A^* mice at disease end-stage, and were associated with a reduced number of dopaminergic neurons in the substantia nigra pars compacta and ventral tegmental area [120]. Peripheral sensory abnormalities, such as axonal swelling and loss, impaired sensory evoked potential, and spinal integration, have been reported both in ALS patients and mice (for review [121,122]). Interestingly, defects in proprioceptive neuron excitability can be observed in brainstem preparation from SOD1 mutant mice at 2 weeks of age [123]. Recent evidence from two different animal models has also shown that the peripheral innervation of spindles by group Ia and II fibers is diminished in the pre-symptomatic stages of disease, although the sensory neuron somata are unaffected at this stage [124,125], and that central synapses are affected only late in the disease process [125]. More in-depth studies exploring spatiotemporal degeneration of long-range neuronal networks will be critical to bridge the communication gap with local networks and therefore provide an integrated view of ALS pathogenesis. Another area for future investigation will be to investigate the functional consequence of the potential loss of other types of glial cells, including ependymal cells, radial glia, satellite cells, and tanycytes.

It may seem surprising that this review does not cover microglia cells, when these cells play an undeniable role in the pathology [126]. To our knowledge, only one study has reported a process of microglial degeneration, known as cytorrhexis [127]. Cytorrhexis, which is characterized by cytoplasmic disintegration, can be observed at the end stage of the disease in the spinal cord of *SOD1^G93A^* rats, although, in the brain stem, nuclear shrinkage can also be observed in shrunken microglial cells. Of note, microglia activation or abnormalities were not observed in the cortex of *SOD1^G93A^* rats. Although cytorrhexis is considered a form of accidental death [128], in contrast to apoptosis, more attention should be drawn to these early results.

In conclusion, the challenge is not only to comprehend the intrinsic elements that make motoneurons selectively vulnerable to the disease, but also to understand: (1) how ALS causative agents specifically affect other neuronal and non-neuronal populations; (2) what is the ordered sequence of cellular events, and their interrelation, that leads to deleterious changes for motoneurons; (3) whether and how the map of brain and spinal connectivity underlies a compensatory homeostatic response; (4) whether the complexity of these non-motoneuronal mechanisms participates in the clinical heterogeneity of the pathology; and (5) how this knowledge can be pertinently anticipated to design new efficient therapies.

## Abbreviations

Aba	Aberrant astrocyte
AbGC	Aberrant glial cell
ALS	Amyotrophic lateral sclerosis
ChAT	Choline acetyl transferase
CPG	Central pattern generator
CNS	Central nervous system
DCA	Dichloroacetate
ER	Endoplasmic reticulum
FUS	Fused in sarcoma
GABA	γ-aminobutyric acid
GFAP	glial fibrillary acidic protein
GLT-1	Glutamate transporter 1
IFNγ	Interferon gamma
iPSC	Induced pluripotent stem cell
MCT	Monocarboxylic acid transporter
mGluR5	Metabotropic Glutamate receptor 5
NPY	Neuropeptide Y
SOD1	Superoxide dismutase-1
SGPC	Spheroid GFAP-positive cell
TRIF	TIR domain-containing adaptor inducing interferon-β
TLR	Toll-like receptor
